# Peripheral Brain-Derived Neurotrophic Factor (BDNF) and Its Regulatory miRNAs as Biological Correlates of Impulsivity in Young Adults

**DOI:** 10.3390/metabo14100529

**Published:** 2024-09-30

**Authors:** Przemyslaw Zakowicz, Beata Narozna, Tomasz Kozlowski, Weronika Bargiel, Maksymilian Grabarczyk, Maria Terczynska, Julia Pilecka, Karolina Wasicka-Przewozna, Joanna Pawlak, Maria Skibinska

**Affiliations:** 1 Collegium Medicum, University of Zielona Gora, 65-417 Zielona Gora, Poland; 2 Center for Children and Adolescent Treatment in Zabor, 66-003 Zabor, Poland; 3 Molecular and Cell Biology Unit, Department of Paediatric Pulmonology, Allergy and Clinical Immunology, Poznan University of Medical Sciences, 60-806 Poznan, Poland; 4 Student’s Research Group “Biology of the Neuron”, Department of Psychiatric Genetics, Poznan University of Medical Sciences, 60-806 Poznan, Poland; 5 Department of Psychiatric Genetics, Poznan University of Medical Sciences, 61-701 Poznan, Poland

**Keywords:** impulsivity, suicide, biomarker, micro-RNA, brain-derived neurotrophic factor (BDNF)

## Abstract

**Background**: Impulsivity assessment may serve as a valuable clinical tool in the stratification of suicide risk. Acting without forethought is a crucial feature in the psychopathology of many psychiatric disturbances and corresponds with suicidal ideations, behaviors, and attempts. **Methods**: We present data on biological and psychological correlates of impulsivity among young adults (*n* = 47). Psychological analysis included both the self-description questionnaire—Barratt Impulsiveness Scale (BIS-11)—and neuropsychological behavioral tests, including the Iowa Gambling Task (IGT), the Simple Response Time task (SRT), and the Continuous Performance Test (CPT). mRNA and micro-RNA were isolated from peripheral blood mononuclear cells (PBMC). Expression levels of Brain-Derived Neurotrophic Factor (BDNF) mRNA and its regulatory micro RNAs, mir-1-3p, mir-15a-5p, mir-26a-5p, mir-26b-5p, and mir-195-5p, were analyzed using the quantitative reverse transcription polymerase chain reaction (RT-qPCR) method. proBDNF and BDNF plasma protein levels were quantified using enzyme-linked immunosorbent assay (ELISA). **Results**: Significant correlations between BDNF mRNA and mir-15a-5p as well as proBDNF levels and mir-1-3p were detected. proBDNF protein levels correlated with motor and perseverance, while mir-26b correlated with cognitive complexity subdimensions of the BIS-11 scale. Correlations between BDNF, miRNAs, and the results of neuropsychological tests were also detected. **Conclusions**: The BDNF pathway shows a clinical potential in searching for biomarkers of impulse-control impairment. BDNF-regulatory micro-RNAs are detectable and related to clinical parameters in the studied population, which needs further research.

## 1. Introduction

As an impulsive act, we define impaired decision style as being without forethought. Impulsivity determines impairment in behavioral regulation and is linked with marked neuropsychological parameters in clinical tests. Until today, impulsivity had been linked to a plethora of psychiatric syndromes, including neurodevelopmental disorders (e.g., attention-deficit hyperactive disorder, ADHD) [1], mood disorders like bipolar disease [2], and personality aberrancies with most prominent borderline personality disorders (BPD) [3]. A broad spectrum of causes meets the common denominator in top-down cognitive controlling deficits and reward processing [4]. Due to Patton’s model of impulsiveness, three different aspects of impairment may be distinguished: lack of planning, motor activation, and attention deficits [5]. These areas of impairment correspond with identifiable patterns of brain activity. 

Regions of interest for impulsivity include both cortical and subcortical structures, supporting the complex interplay between frontal regulatory neural networks and the reward processing dopaminergic system. The upper floor of the putative impulse control brain network consists of the inferior frontal gyrus (IFG) and the pre-supplementary motor area (p-SMA); regarding the subcortical level, the ventral striatum has been indicated as crucial. A recent meta-analysis by Kaiser et al. also suggests developmental trajectory patterns towards the modification of reward-sensitivity mediated by the ventral striatum, stressing differences between the adolescent period and adulthood [6]. Misbalance between the development of subcortical regions (steering the mood, reward prediction, and drive) and prefrontal maturation makes young people highly vulnerable to impulsivity.

Impulsivity is also perceived as the result of neurotransmission imbalance. Besides the pivotal role of dopaminergic transmission [7], recent data also suggest the emergent role of glutamatergic signaling. This approach was coined due to studies on psychostimulant abuse research and tries to elucidate the phenomenon of psychological reinforcement after drug intoxication, linking it with learning strategies and cognitive plasticity [8]. Glutamate is a neurotransmitter engaged in the excitatory–inhibitory balance in neural networks [9]. The ability of neural networks to adapt to changes in the environment depends on synaptic plasticity patterns and the shaping of new connections. Such a state implicates an individual’s adaptive behavior regarding stress response, hence the putative role of glutamatergic synaptic plasticity in the learning resilience mechanisms [10]. 

Synaptic plasticity links with molecular pathways engaged in neuronal growth and connection forming [11]. The need to track neurotrophin aberrancies among impulsive individuals seems to have diagnostic potential in the identification of particular objective biomarkers. From those with a high clinical potential, the brain-derived neurotrophic factor (BDNF) reveals a biomarker’s potential, e.g., in impulsive behavior, mood disorders and the schizophrenia-spectrum [12,13,14]. BDNF serves as the metabolic hub for synaptic plasticity, mediating neuronal growth, forming novel connections, and synaptic homeostasis [15]. Recent data suggest its pivotal role in the modulation of neuronal death, which may be an antecedent to drug-induced psychosis [16]. Thus, the BDNF-pathway seems to be a biological grip for synaptic dysregulation [17]. 

The presence of BDNF was evidenced in peripheral blood plasma and serum, and correlates with the clinical picture and treatment response in psychiatric disorders. BDNF was also linked to impulse-control disorders; functional polymorphism of BDNF rs6265 (Val66Met) was indicated as significant for attentional impulsivity in methamphetamine abusers [18]. The marked impact of rs6265 was also studied for impulsiveness levels in bipolar disorder (BD) [19]. Similarly, serum BDNF levels had been recently suggested as corresponding with the rate of impulsivity in a cohort of patients [13]. The altered expression of neurotrophins, including BDNF, was also recently studied in the context of micro-RNA (miRNAs). We define miRNAs as short, non-coding, and about 22 nucleotide fragments of RNA. The cellular role in gene expression is based on post-transcriptional regulation [20]. MiRNAs are detectable peripherally in physiological fluids like blood serum and cerebrospinal fluid; hence, neuronal-specific miRNAs were recently suggested as potential biomarkers of neuropsychiatric disorders, including neurodegeneration and psychosis [21]. The engagement of miRNAs in the processes of neurotrophin synthesis determines its crucial role in synaptic plasticity, neuronal growth, and the resilience of neural networks. miRNAs can directly or indirectly interfere with BDNF expression [20]. 

Linking biological marker studies with clinical and behavioral tests provides multifactorial analysis for screening tools for impulse-control disorders. Routinely, behavioral tests assessing impulsivity and decision-making based on win/loss gambling tasks (e.g., Iowa Gambling Task) [22] measure the ability of stimulus–response inhibition (like in the case of a simple reaction time task [23]) or require a proper response on presented targets during continuous attention tasks (continuous performance task [24]). The second aspect of impulsivity assessment includes clinical tests of patient self-assessment, like the Barratt Impulsiveness Scale [5] and Eysenck Impulsiveness Inventory [25]. In the Barratt Impulsiveness Scale, the determination of impulsivity requires a 30-item rating regarding 6 primary factors: attention, motor impulsiveness, self-control, cognitive complexity, perseverance, cognitive instability, and 3 secondary dimensions: attentional impulsiveness, motor impulsiveness, and non-planning impulsiveness. 

Here, we present a study among a healthy young adult group regarding both parameters of impulsiveness (computerized neuropsychological tests: Iowa Gambling Task (IGT), Simple Response Time task (SRT) test, and Continuous Performance Task (CPT), and self-description Barratt Impulsiveness Scale BIS-11) and potential biomarkers from the peripheral venous blood related to BDNF expression. Biological analysis encompassed peripheral BDNF mRNA expression, proBDNF and BDNF plasma protein levels, and BDNF’s regulatory miRNA (mir-1-3p, mir-15a-5p, mir-26a-5p, mir-26b-5p, mir-195-5p) levels. miRNAs selected in this work in various studies have shown a modulating effect on BDNF expression through attachment to the 3’-UTR of mRNA and inhibition of translation [26,27,28,29]. We aimed to track the correlations between biological factors and measures of impulsive behavior. The presented study is part of a broader project aiming to find biological correlates of suicide risk. Growing interest on suicidality prevention among vulnerable individuals sets the question about proper screening and targeted prophylaxis. The global burden of suicidal acts reaches over 700,000 people yearly; suicide is also one of the leading cause of death among young adults (15–29 years old) [30]. From this point of view, impulsivity appears as the easily measured personality dimension with clinical utility. 

## 2. Materials and Methods

### 2.1. Participants

The study involved 40 healthy young adults aged 19–30, including 17 females, with a mean age of 23.3 (SD 2.9), and 23 males, with a mean age of 24.9 (SD 3.40). The majority of the participants were medical students (*n* = 32), and 15 subjects had finished higher education and were employed. Exclusion criteria were any psychiatric diagnosis, first-degree relatives with psychiatric diseases, severe somatic or neurological disorders, psychoactive substance misuse, pregnancy, or breastfeeding. All participants were of Caucasian origin, recruited from May 2022 to December 2022 at the Department of Psychiatric Genetics, Poznan University of Medical Sciences, Poland. All participants gave written informed consent. The study was conducted according to the Helsinki Declaration. The Ethics Committee at the Poznan University of Medical Sciences approved the study protocol (permission no 39/22).

### 2.2. Impulsivity Assessment

The self-rated Barratt Impulsiveness Scale (BIS-11) was applied. The items are scored on a four-point Likert scale, with higher total scores indicating higher trait impulsivity. According to Barratt, impulsiveness includes three major dimensions: attentional impulsivity (tendency to change attention quickly and difficulties with deferring gratification), non-planning impulsivity (focusing on the present moment without thinking about the future), and motor impulsivity (acting without preemptive thinking). Each of these three major dimensions of BIS-11 consists of two subdimensions: attentional impulsivity—attention and cognitive instability, and motor impulsivity—motor and perseverance and non-planning impulsivity with self-control and cognitive complexity [31].

Computerized versions of the Iowa Gambling Task (IGT), Simple Response Time task (SRT) test, and Continuous Performance Task (CPT) were applied to measure the impulsive behavior of participants. All computer tests were performed in the same order: SRT, CPT, and IGT between 9:00 a.m. and 12:00 p.m. with the use of the Experiment Building Language (PEBL) battery [32].

### 2.3. Biological Analyses

Five milliliters of venous blood was drawn into Vacutainer^®^ K2EDTA tubes (cat. No 367864) between 07:30 and 09:30 after overnight fasting. A total of 400 uL of whole blood was transferred into 1.5 mL Eppendorf tubes, thoroughly mixed with 400 uL of Lysis Buffer (DL buffer, NucleoSpin RNA blood, cat. No 740210.50, Machery-Nagel, Düren, Germany), and stored at −80 °C until extraction. Whole RNA extraction from the blood procedure was performed using a NucleoSpin RNA blood kit, strictly according to the manufacturer’s instructions. Concentration and quality of extracted RNA was measured using a Nanodrop 2000 spectrophotometer (ThermoFisher Scientific, Wilmington, DE, USA). 

Reverse transcription (RT) reaction of total mRNA was performed using GoScript™ Reverse Transcription System (cat. No A5000, Promega Corp., Madison, WI, USA), and BDNF mRNA quantitative PCR (qPCR) reaction was conducted using GoTaq^®^ qPCR Master Mix (cat. No A6001, Promega Corp., Madison, WI, USA). mBDNF primer sequence was as follows: BDNF forward, 5′-CTACGAGACCAAGTGCAATCC-3′; BDNF reverse, 5′-AATCGCCAGCCAATTCTCTTT-3′ [33]. Beta(2)-microglobulin served as an endogenous control for qPCR reaction, with primer sequence as follows: B2M forward, 5′-CACCCCCACTGAAAAAGATG-3′; B2M reverse, 5′-ATATTAAAAAGCAAGCAAGCAGAA-3′. 

RT and qPCR reaction of mir-1-3p, mir-15a-5p, mir-26a-5p, mir-26b-5p, and mir-195-5p, with mir-451a as an endogenous control were performed using a TaqMan^®^ Advanced miRNA cDNA Synthesis Kit (cat. No A28007, ThermoFisher Scientific, Wilmington, DE, USA) and TaqMan^®^ Advanced miRNA Assays (cat. No, accordingly: 477820_mir, 477858_mir, 477995_mir, 478418_mir, 477957_mir, 478107_mir, ThermoFisher Scientific, Wilmington, DE, USA), according to the manufacturer’s instructions. RT and qPCR reactions were performed using an Eppendorf Realplex2 Mastercycler (Eppendorf, Oldengurg, Germany) and a 7900HT fast-real time PCR system (Applied Biosystems^TM^, Waltham, MA, USA). proBDNF and BDNF plasma quantifications were performed as described previously [34].

### 2.4. Statistical Analyses

BDNF mRNA and miRNA expression was analyzed with DataAssist software v.3.01 (ThermoFisher Scientific). The comparative CT method [35] was used for calculating relative quantification of gene expression. 

The Kolmogorov–Smirnoff test was used to check the normality of the data. Most studied clinical and biological variables showed non-normal distribution; thus, nonparametric methods were used. Mann–Whitney U-test and Spearman rank correlation was applied. Non-parametric statistics are less prone to outlier values than parametric ones. We did not intend to remove outlier values while they are observed in biological factors—proteins and mRNA or miRNA levels. The significance level was set at *p* < 0.05. A power of ≥80 with the study group of *n* = 40 is achieved with R ≥ 0.43 in the correlation analysis (https://sample-size.net/correlation-sample-size/, accessed at 18 September 2024). Strength of correlation is based on correlation coefficient R parameter, with following values indicating: 01–03—weak, 0.4–0.6 moderate, and 0.7–0.9 strong correlation [36]. The statistical analyses were performed using Statistica v13 software (StatSoft, Krakow, Poland). 

## 3. Results

Mean values (SD) and comparison of age and biological parameters between female and male participants are presented in Table 1. We did not observe any differences concerning gender for age, the expression levels of BDNF mRNA and miRNA (mir-1-3p, mir-15a-5p mir-26a-5p, mir-26b-5p, mir-195-5p), proBDNF and BDNF plasma protein levels.

**Figure 1:**
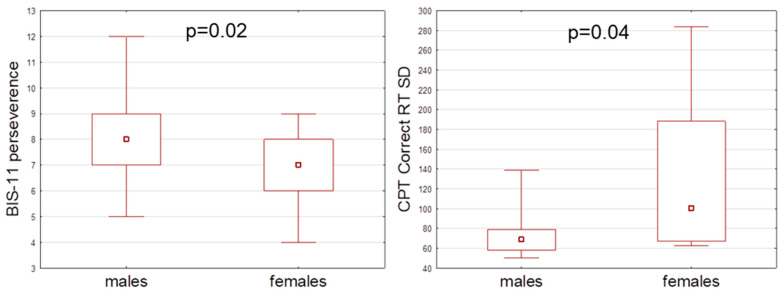
Barratt Impulsiveness Scale (BIS–11) perseverance subdimension and Continuous Performance Task correct RT SD comparisons between males and females.

We detected higher BIS-11 perseverance (Z = 2.45, *p* = 0.02) in males (Figure 1). In the Continuous Performance Task (CPT), females presented higher correct RT SD parameters compared to males (Z = −2.08, *p* = 0.04) (Figure 1). We did not observe any significant differences in measured parameters regarding sex for the Iowa Gambling Task (IGT) and Simple Response Time task (SRT). 

### 3.1. Correlations of Biomarkers

Spearman correlation rates revealed significant correlations at moderate and strong relationship levels for mir15a-5p and BDNF mRNA (R = 0.41, *p* < 0.01), mir195-5p (R = 0.32, *p* = 0.05), mir26b (R = 0.62, *p* < 0.01), mir26a (R = 0.6, *p* < 0.01), negative correlations between mir1-3p and plasma proBDNF (R = −0.34, *p* = 0.03), mir195-5p and mir26b (R = 0.48, *p* < 0.01)/mir26a (R = 0.45, *p* < 0.01). For mir26a and 26b, we received a strong correlation rate (R = 0.7, *p* < 0.01). No other correlations between analyzed biological factors were found. Results of significant correlations between biological factors are presented in Table 2.

### 3.2. Correlation Analysis of BIS-11 Scores and Neuropsychological Tests Results

Spearman rank order correlation rates reached significance between BIS-11 values in the studied population and some parameters in behavioral tests. Most relevant correlations were achieved for the Iowa Gambling Task (IGT) and the Continuous Performance Task (CPT). In the Iowa Gambling Task (IGT), BIS-11 cognitive complexity and perseverance correlated negatively with the number of risky (AB) cards (for both domains, R = −0.39, *p* = 0.04). The number of safe “D” cards (nD) in the test performance correlated with the non-planning domain in BIS-11 (R = 0.47, *p* = 0.01) and self-control (R = 0.42, *p* = 0.02). 

In CPT, we observed significant correlations between BIS-11 non-planning and total errors (R = 0.47, *p* = 0.02), and between cognitive complexity and error response time (R = 0.46, *p* = 0.02). 

### 3.3. Correlations of Biological Factors and Impulsivity 

We observed correlations between proBDNF plasma levels and BIS-11: motor impulsivity score (R = 0.31, *p* = 0.04) and negative correlations for the perseverance score (R = −0.32, *p* = 0.05). BIS-11 cognitive complexity was also negatively (R = −0.32, *p* = 0.04) correlated with mir-26b-5p expression.

Regarding SRT, we obtained significant correlations between BDNF protein levels and correct response time min (R = −0.41, *p* = 0.04), mir-1-3p and median correct response time (R = −0.44, *p* = 0.03), and mir-26a and correct response time min (R = 0.43, *p* = 0.04).

CPT correct targets were negatively correlated with BDNF protein levels (R = −0.43, *p* = 0.03). mir-1-3p negatively correlated with correct response time: mean (R = −0.42, *p* = 0.03), median (R = −0.40, *p* = 0.04), and SD (R = −0.55, *p* < 0.01); mir-26b with CPT error response time SD (R = −0.53, *p* = 0.005); and mir-15a-5p with CPT error response time SD (R = −0.41, *p* = 0.04). We did not observe any relevant correlations for any other biomarkers in the continuous performance task parameters. 

For the Iowa Gambling Task, we obtained significant correlation of biological parameters and risky/non-risky choices. BDNF protein levels negatively correlated with risky nB1 choices (R = −0.39, *p* = 0.04) and positively with non-risky nC1 (R = 0.5, *p* = 0.007). mir-15a-5p positively correlated with risky choices: nB (R = 0.42, *p* = 0.02), nAB (R = 0.4, *p* = 0.04), nB5 (R = 0.4, *p* = 0.04), and negatively with non-risky choices: nCD(R = −0.4, *p* = 0.04) and nCD-nAB (R = −0.4, *p* = 0.04). Positive correlation with risky choice nB5 showed mir-26b (R = 0.4, *p* = 0.03) and mir-26a (R = 0.42, *p* = 0.02). mir-26b also negatively correlated with non-risky nC (R = −0.46, *p* = 0.01) and nC5 (R = −0.4, *p* = 0.04)

## 4. Discussion

Linking the self-assessment and behavioral tests with objective blood-derived biomarker analysis may lead to increased sensitivity in diagnosis. In this study, we aimed to preliminary check the potential value of BDNF expression pathway factors as suitable for such analysis. To the best of our knowledge, this is the first study examining peripheral BDNF expression on mRNA and protein (proBDNF and mature BDNF) levels, along with its regulatory microRNAs in correlation with impulsivity in healthy young adults.

High hopes linked to BDNF as a valuable biomarker led to many research papers showing this protein as non-specific to particular neuropsychiatric diseases. An aberrant profile of BDNF expression was, up to date, connected with depressive episodes, bipolar disease, or psychotic states, which suggests a modulatory role of BDNF in neural network pathologies rather than a restricted type of biomarker [12,13,14]. Hence, the BDNF expression pathway studies, which include regulatory miRNA, may be the source of valuable data [20]. From the clinical point of view, the useful direction may be resigning from clinical diagnosis/healthy control comparisons in psychiatry until we have a well-grounded biological definition of each psychiatric state. Focusing on a specific symptom, e.g., impulsivity, may help to define a more homogenous study group, giving more reliable biological insights.

Impulsivity seems to be a trans-diagnostic symptom found in personality disorders, attention deficit hyperactive disorder (ADHD), or bipolar disease, and may be treated as a separate factor of suicidal vulnerability (see our previous study [2]). Impulsivity may be considered as the result of direct cortico-subcortical dysfunction, both inherited and acquired during disease progression, like in the case of dementia. Defined neurobiology may give a defined biomarker, but the history of BDNF studies shows how to not focus on simple concentration/expression profiles. Available data suggest preferably to focus on expression misbalance as the molecular source of pathology. The first interplay in the BDNF pathway was noticed between proBDNF and BDNF, where opposite roles were postulated; in the second phase of studies, the underlined interplay was referred to the p75NTR/TRKB receptor acting in pro/anti-apoptotic states [37]. In our study, we tried to suggest that misbalanced items may be detected on earlier phases of the BDNF pathway; the presented results showed the significant correlations of miRNA levels in peripheral blood for both the self-description questionnaire and neuropsychological behavioral tests. The correlational model of this analysis served firstly to track the relationship between different miRNAs in the peripheral blood and secondly to find the co-occurrence of miRNA levels in the clinical picture. 

We did not detect a correlation between BDNF mRNA expression in PBMC and proBDNF or BDNF plasma protein levels in the presented study. Opposite results, showing a strong correlation between BDNF mRNA expression and BDNF plasma protein levels in healthy persons, were shown in the recent research by Sokolowski et al. (2023) [38]; however, the study was performed on older participants (mean age 54 years) compared to our group. Additionally, no proBDNF plasma levels were determined. The serum BDNF protein level was correlated with mRNA expression in the other study [39]. Considering that no correlation between plasma and serum BDNF protein levels were detected [40] and BDNF release from platelets during clotting was observed, difficulties in the interpretation of the results remain.

In our study, BDNF mRNA levels positively correlated with mir-15a-5p, which is in opposition to other studies, where mir-15a-5p has been shown to be responsible for the inhibition of BDNF expression [20]. Plasma proBDNF level in our group negatively correlated with mir-1-3p, which was shown to down-regulate BDNF expression [41]; however, there are no available data connecting proBDNF and mir-1-3p.

We noticed positive correlations between all of the studied miRNAs except mir-1-3p. Regarding mir-195-5p, preclinical data from H4 neural cells suggest suppressive activity of mir-195-5p on BDNF expression [42]. Other preclinical studies on animal models of alcoholism (Long–Evans rats) targeted the peripheral level of mir-195-5p as related to the early-onset heavy style of alcohol addiction [43]. As Ehinger et al. (2021) suggested, mir-195-5p is engaged in neural cell synaptogenesis and cytoskeleton development, which links with synaptic plasticity [43]. mir-195-5p was also tested in the study cohort with schizophrenia diagnosis [44]. In relation to the healthy control, the schizophrenia group exhibited elevated mir-195-5p in peripheral blood, with a negative correlation regarding BDNF [44]. However, the regulatory role of this particle remains not fully elucidated; mir-195-5p seems to be a valuable source of further investigation. 

The literature on mir-15a-5p is sparse, suggesting its upregulation in focal cortical dysplasia (FCD) patients’ peripheral blood [45]. As the authors suggested, the role of mir-15a-5p may lead to altered gene expression, intracellular transmission, and aberrant neurons’ homeostasis leading to epileptogenesis [45]. The engagement of mir-26a and mir-26b (mir26s) in executive control may be connected with data on functional Val66Met BDNF polymorphism and executive worsening [46]. In the in silico study cited above by Caputo et al. (2011), mir26s may exhibit high affinity to the 3′UTR region, which contains single nucleotide polymorphisms (rs11030100 and rs11030099), being in high linkage-disequilibrium with Val66Met [26]. The role of mir-26s seems to be controversial in this manner and needs further explanation. We did not find any data in the literature on mir-1-3p engagement in both animal and human models in central nervous system research. Identified data in the literature review suggest its altered activity in peripheral blood after physical exercise, which was hypothetically linked to antidemential immune-neural system homeostasis [47]. mir-1-3p may also interrupt the cell cycle in neuroendocrine cells in preclinical models due to the cyclin-dependent kinase 4 (CDK4) pathway [48]. 

Regarding correlations of miRNAs and clinical data, we observed significant correlations from self-assessment and behavioral tests. For BIS-11, correlations were detected between cognitive complexity and mir-26b. The Barratt Impulsiveness Scale (BIS-11) remains the most widely used screening tool for assessing impulse control and decision-making style [5]. BIS-11 was evidenced in conducted studies as highly correlated with biological parameters, like brain surface structure [49], genetic variants [50], or protein biomarkers [51]. Results of our study suggest the existing molecular background of BIS-11 assessment in the context of BDNF expression and brain plasticity. 

In this article, we also tried to combine results stemming from the PEBL battery of neuropsychological measurement and miRNA levels. The most emergent miRNAs correlated with executive performances were mir-26a, mir-26b, mir1-3p, and mir-15a-5p. All of the studied miRNAs were classified as pro-impulsive in our model due to the correlation with risky decision-making style and the negative relationship with correct response parameters in behavioral tasks. The parameters of the Iowa Gambling Task reflect the shaping of decision-making in studied subjects [22]. mir-15a-5p exhibited significant correlations with risky decision style, like a higher number of risky “A” and “B” cards and a higher number of risky cards (“B” deck) in the last 20 choices of the task. Similar pro-risky correlations were obtained for both mir-26a and mir-26b. Here, we also noticed strong negative correlations between mir26s levels and SRT/CPT correct response time parameters, also interpreted as pro-impulsive. 

Our study detected correlations of proBDNF protein plasma levels with impulsivity domains of the BIS-11 scale and BDNF with neuropsychological impulsivity measures. proBDNF correlated positively with motor impulsivity scores and negatively with perseverance scores. The BDNF level correlated negatively with correct response time in SRT and correct targets in CPT. In IGT, we detected a negative correlation between BDNF and risky choices nB1 and a positive correlation between BDNF and non-risky choices nC1. Pasyk et al. (2020) found a positive correlation between BDNF serum levels and BIS-11 total scores in patients diagnosed with mood, psychotic, anxiety, or substance use disorders with a low risk of suicide [13]. High BDNF serum levels correlated with high impulsiveness in post-traumatic stress disorder in the study by Martinotti et al. (2015) [52]. proBDNF has the opposite effect to BDNF. Thus, it is difficult to compare our results, considering that protein measures were performed in plasma in our study, which can substantially influence results. Heterogenous results of analysis of BDNF serum levels and IGT results were reported in gambling disorders [53], eating disorders [54], and schizophrenia [55], with an emphasis on net scores (total or partial). Our results show consistent correlations of low BDNF with risky and high BDNF with non-risky choices in IGT, indicating lower impulsivity associated with higher BDNF levels. No studies on healthy populations and plasma proBDNF and BDNF levels concerning impulsive behavior were published previously. This preliminary study gives an opportunity to use more in-depth biological studies in the BDNF pathway and should be further tested in clinical populations. Creating an independent biological marker of impulse-control impairment may be used in the future as a reliable screening tool and targeted prophylaxis. 

## 5. Conclusions

The presented study sheds some initial light on the potential role of BDNF-linked miRNAs and their clinical utility. This study shows the ability of BDNF regulatory mi-RNAs. To our knowledge, this is the first paper describing miRNAs in such a context, highly exploring available data. The main limitations of the study are: (i) the low sample size is insufficient to draw the definitive clinical conclusions, (ii) the cross-sectional model of this study does not provide data on the causal relationship between biomarkers and impulsivity. Despite the potential value, results of this research should be interpreted carefully in a preliminary and proof-of-concept manner; future studies should involve larger cohorts and more biological factors involved in the regulation of BDNF expression. 

## Figures and Tables

**Table 1:** Mean values of age, ΔCT: mRNA BDNF, mir-1-3p, mir-15a-5p, mir-26a-5p, mir-26b-5p, mir-195-5p; proBDNF, BDNF plasma protein levels.

	Whole Population (*n* = 40)		Females (*n* = 17)		Males (*n* = 23)		
	Mean	SD	Mean	SD	Mean	SD	*p* *
Age	24.28	3.27	23.35	2.89	24.96	3.42	0.17
mRNA_BDNF	33.60	3.26	32.95	2.86	34.08	3.51	0.22
mir 1-3p	5.60	1.83	5.25	2.14	5.86	1.57	0.29
mir 15a-5p	3.11	3.11	3.59	4.36	2.75	1.73	0.75
mir195-5p	8.00	2.21	8.04	2.06	7.96	2.37	0.77
mir26b	3.48	1.70	3.25	1.73	3.65	1.69	0.48
mir26a	3.74	1.55	3.50	1.46	3.92	1.62	0.52
proBDNF (pg/mL)	4815.68	9700.31	7695.39	14307.44	2687.19	2605.29	0.12
BDNF (pg/mL)	1820.09	2108.88	1562.89	2057.55	2010.20	2171.67	0.42

* Mann–Whitney U test.

**Table 2:** Spearman’s correlations of biological factors: BDNF mRNA, proBDNF and BDNF plasma protein levels, mir-1-3p, mir-15a-5p, mir-26a-5p, mir-26b-5p, mir-195-5p.

Biological Factors	R	*p*
mRNA_BDNF and mir-15a-5p	0.416	0.008
proBDNF and mir-1-3p	−0.336	0.034
mir-15a-5p and mir-195-5p	0.319	0.047
mir-15a-5p and mir-26b	0.622	0.000
mir-15a-5p and mir-26a	0.595	0.000
mir-195-5p and mir-26b	0.479	0.002
mir-195-5p and mir-26a	0.451	0.004
mir-26b and mir-26a	0.697	<0.000

## Data Availability

The original contributions presented in the study are included in the article; further inquiries can be directed to the corresponding authors.
